# The Means by Which the Temperature of the Body Is Maintained in Health and Disease

**Published:** 1897-07-10

**Authors:** W. Hale White

**Affiliations:** Physician to, and Lecturer on Pharmacology at, Guy's Hospital


### Medical Progress and Hospital Clinics.

[*The Editor will be glad to receive offers of co-operation and contributions from members of the profession. All letters should be addressed to* The Editor, at the Office, 28 & 29, Southampton Street, Strand, London, W.C.]

### THE MEANS BY WHICH THE TEMPERATURE OF THE BODY IS MAINTAINED IN HEALTH AND DISEASE.

The Croonian Lectures, delivered before the Royal College of Physicians of London.

By W. Hale White, M.D., F.R.C.P. Lond., Physician to, and Lecturer on Pharmacology at, Guy's Hospital.

### Lecture IV.

A third method which had been used in the study of the amount of heat produced under different conditions was the chemical. This depended on the well-known fact that the breaking down of any chemically complex substance led to the liberation of energy, and that by comparative experiments it was possible to calculate the amount of energy which would be set free by the breaking down of the substances taken as food. There were many difficulties, however, in calculating the heat production of an animal from its diet. In the first place we did not know what proportion of any meal was absorbed from the gastro-intestinal tract; secondly, all the energy set free in the body was not manifested as heat, some taking the form of mechanical work, a very variable and uncertain quantity; and thirdly, the food might be stored up in the body and not broken down for some time. It was possible, however, to get at the matter in another way, viz., by observing the amounts of nitrogen and carbon excreted and calculating from them the quantity of proteid, fats, and hydro-carbons which must have been broken down to produce these quantities, and then calculating how many calories of heat would be produced by the decomposition involved.

In every one of these methods there were many elements of uncertainty, so many that one could not but be surprised to find how nearly the results arrived at by different methods tallied with one another.

A description was given of the investigations made by May, who came to the conclusion that in febrile pyrexia the excretion of $C O_2$ was not much increased, and that the brunt of the destruction fell on the proteids. He used rabbits, in whom he induced swine erysipelas. For some days before the production of this fever they were starved, so as to get their excretion of urea steady; and during the fever May most carefully observed the output of nitrogen and carbon, both in the urine and the breath, for more than 20 hours at a time, and he calculated what must have been the original amount of proteid and fat tissue which would, when broken down, correspond to the nitrogen and carbon he found excreted. Using then the heat values of proteid and fat which had been worked out by Stohmann, he was able to estimate the number of calories of heat which would be set free by the destruction of these amounts of proteids and fat. The result of his experiments was to show distinctly that when a starving rabbit was suffering from swine erysipelas, there was an increased heat production, and that this was due entirely to an increased destruction of proteid.

The next experiments of importance were those which showed how the administration of sugar saved the destruction of albumen and fat which went on in starvation, and then came those which showed that the administration of sugar effected a similar saving in fever. Experimental medicine thus showed why milk and farinaceous diet was the best for fever, as clinical medicine had found by experience was the case. So far as it was possible to check these experiments by observations upon man, everything pointed to their holding good as to him also, for they went to show that in the course of pyrexia the amount of $C O_2$ excreted was not increased.

The experiments which Dr. Hale White had made upon rabbits in whom pyrexia had been induced by cerebral puncture, also showed only very slightly increased output of $C O_2$.

Many experiments were described which had been made on hibernating animals, the result being to show that the rise of temperature which occurred when hibernating dormice become active was unlike the rise of temperature which occurred when a rabbit was afflicted with swine erysipelas or had its temperature raised by damage to the corpus striatum, for it was accompanied by an enormous increase in the output of carbonic acid. This went to show that the metabolic processes of the pyrexia due to cerebral lesions, and of that due to fever, were quite different from those of health, and not merely an exaggeration of these.

The three main conclusions arrived at then were: 1. In man at least pyrexia is not produced by the same method in all fevers; sometimes the rise of temperature is due to an increased production of heat, and sometimes to a diminution of the loss. 2. In the present state of our knowledge animal calorimetry is very difficult, and the results obtained from calorimeters must be received very guardedly. 3. In some forms of pyrexia in which the production of heat is increased the metabolism leading to this takes place in the proteid tissues of the body.

A very important question then arose as to how far pyrexia in itself was harmful or beneficial. Why was it that almost all the micro-organisms which were harmful to the body raised its temperature? Could it be that pyrexia, like phagocytosis or chemiotaxis, was a defensive mechanism, and was in some way harmful to the infective organisms? Of course, it did not follow from this view that the higher the temperature of the body the better the prognosis, for the higher temperature might be taken to indicate that the dose of infection was very severe, and that therefore the body did all it could to resist the invasion, nor, on the other hand, would it follow that if the temperature did not rise much the dose of infection was slight, for it might be that the body was feeble and had but little power of raising its temperature and therefore defending itself. But Dr. Hale White mentioned that in a case of typhoid fever which he had seen, in which during the second week the temperature had been very irregular, the patient always felt better and had a better pulse when her temperature was high than when it was low. A few years ago much had been hoped from the use of antipyretic drugs in the treatment of fever, but experience had led to the conclusion that as a general rule it did more harm than good to directly treat the pyrexia in such cases.

Some few fevers were treated by agents which lowered the temperature, but these very fevers were instances of the exception proving the rule. When ague was treated by quinine, the temperature fell and the patient was better. This, however, was not because the quinine was an antipyretic, but because it was a poison to the plasmodium malariæ; the patient's temperature fell because the pyrexial agent was destroyed. The same also was true in regard to syphilis.

In regard to cold bathing in typhoid fever, it must be remembered that it acted in other ways besides lowering the temperature, especially in the direction of increasing the elimination of toxins.

Experiments had been made, the tendency of which was to show that animals, in whom an artificial pyrexia had been induced by puncture of the corpus striatum, resisted a dose of infection which was fatal to others.

There was, then, much reason to believe that the pyrexia of a fever was a protective mechanism.

